# Partial Altitudinal Migration of a Himalayan Forest Pheasant

**DOI:** 10.1371/journal.pone.0060979

**Published:** 2013-04-26

**Authors:** Nawang Norbu, Martin C. Wikelski, David S. Wilcove, Jesko Partecke, Ugyen, Ugyen Tenzin, Sherub, Tshering Tempa

**Affiliations:** 1 Ugyen Wangchuck Institute for Conservation and Environment, Lamai Gompa, Bumtang, Bhutan; 2 Max-Planck Institute for Ornithology, Radolfzell, Germany; 3 International Max-Planck Research School for Organismal Biology, University of Konstanz, Konstanz, Germany; 4 Woodrow Wilson School, Princeton University, Princeton, New Jersey, United States of America; 5 College of Forestry and Conservation, University of Montana, Missoula, Montana, United States of America; Utrecht University, The Netherlands

## Abstract

**Background:**

Altitudinal migration systems are poorly understood. Recent advances in animal telemetry which enables tracking of migrants across their annual cycles will help illustrate unknown migration patterns and test existing hypotheses. Using telemetry, we show the existence of a complex partial altitudinal migration system in the Himalayas and discuss our findings to help better understand partial and altitudinal migration.

**Methodology/Principal Findings:**

We used GPS/accelerometer tags to monitor the migration of Satyr tragopan (*Tragopan satyra*) in the Bhutan Himalayas. We tagged 38 birds from 2009 – 2011 and found that tragopans are partially migratory. Fall migration lasted from the 3^rd^ week of September till the 3^rd^ week of November with migrants traveling distances ranging from 1.25 km to 13.5 km over 1 to 32 days. Snowfall did not influence the onset of migration. Return migration started by the 1^st^ week of March and lasted until the 1^st^ week of April. Individuals returned within 4 to 10 days and displayed site fidelity. One bird switched from being a migrant to a non-migrant. Tragopans displayed three main migration patterns: 1) crossing multiple mountains; 2) descending/ascending longitudinally; 3) moving higher up in winter and lower down in summer. More females migrated than males; but, within males, body size was not a factor for predicting migrants.

**Conclusions/Significance:**

Our observations of migrants traversing over multiple mountain ridges and even of others climbing to higher elevations is novel. We support the need for existing hypotheses to consider how best to explain inter- as well as intra-sexual differences. Most importantly, having shown that the patterns of an altitudinal migration system are complex and not a simple up and down slope movement, we hope our findings will influence the way altitudinal migrations are perceived and thereby contribute to a better understanding of how species may respond to climate change.

## Introduction

Animal migration is a complex phenomenon exhibited across many taxonomic groups [1], [2] and has been a subject of study for decades due to its prevalence across taxa and its importance in the life history of organisms [3]. Migration as a tactic also elucidates mechanisms by which organisms interact with their environment [4] and as such is important in understanding an organism's response to its environment [5]. However, given that the phenomenon of migration is increasingly under stress [2], [6], [7], it is important to better understand aspects and systems which have not yet received adequate study.

Bird migration in particular has received a great deal of attention from biologists [8], [9]. In addition to long distance, cross continental latitudinal movements, many birds also undertake annual migrations along elevational gradients in montane environments [10]. So far, only a few studies have focused on altitudinal migration systems [11]–[25] and have mostly been viewed as individuals moving from higher elevations to more favorable lower elevations and vice-versa in response to fluctuating environmental conditions such as availability of food [14], [18]–[21], changes in weather [22], or trade-offs between survival and predation risks [23]. However, very few studies have examined these in detail [11], [12], [14], [15], [17]; and very little is known about altitudinal migration patterns (but see [24] and [13], [25]).

It has been suggested that most migrations may in fact be partially migratory systems [26], where only a fraction of the population migrates [27], [28]. This has been found to be true also for altitudinally migrating tropical birds [16], [22], [29], [30]. In such cases, where a population is partially migratory, emphasis has been placed on determining differences between migrants and non-migrants [29], [31], [32]. Three main hypotheses have been used to explain partial migration (albeit these hypotheses were initially formulated to explain differential migration): the dominance hypothesis [33], the body-size hypothesis [34], [35], and the arrival time hypothesis [34]. Here, we consider only the body size and the arrival time hypotheses, given difficulties of measuring dominance in the field.

The body size hypothesis predicts that smaller individuals will migrate once food availability declines or due to intolerable colder temperatures. Here, two main mechanisms are invoked. One is food availability, where it is assumed that bigger individuals would be better able to compete for food or have greater fasting ability during times of food scarcity. The other mechanism is the ability to withstand temperature changes, where larger individuals, who have lower surface area to volume ratio, are better able to withstand colder temperatures and therefore remain sedentary (or migrate if it becomes too hot [36]). This differential ability to thermo-regulate based on size has also been stated as the thermal tolerance hypothesis [37]. Most studies in the temperate zone tend to support the body size hypotheses [38]. However, recent studies from the neo-tropics [29], [39] have shown that bigger individuals are more likely to migrate, highlighting the need to re-interpret prevailing hypotheses based on site and social system specific conditions (reviewed by [26]).

The arrival time hypothesis [34] posits that individuals which establish territories during the breeding season will be less likely to migrate. The hypothesis further predicts that in cases where territorial individuals do migrate, they migrate shorter distances than individuals who do not need to establish territories (i.e., females in our case) resulting in differential migration. Availability of food and declining temperatures at limited breeding grounds are invoked as the underlying driving mechanisms.

Studies so far offer mixed support for the current hypotheses [26]. Recent telemetry techniques (e.g., gps-tracking) which allow researchers to track animals throughout their annual cycle may enable better testing of extant hypotheses and also illustrate hitherto unknown patterns of migrations. Unfortunately, most altitudinal migration studies so far have used telemetry only to a limited extent [11]–[15], [24], [25] even though these systems occur over comparatively smaller geographic space and are therefore more feasible study systems. In addition to contributing to a better understanding of migration ecology, telemetry data are also crucial to ensuring the adequate conservation of species that undertake such migrations [13]. Since mountain systems will witness faster rates of warming [40], altitudinal migration systems across the world's mountains [41] may be affected in novel and unpredictable ways [42]. Most species of birds in the Himalayas (including Bhutan) are considered to be altitudinal migrants [43]. Yet, to our knowledge, there are no studies on altitudinal migration of birds employing telemetry in the Himalayas.

We used state-of-the-art GPS/accelerometer tags to monitor migration in a high altitude pheasant, the Satyr tragopan (hereafter referred to as tragopan/s) in the Bhutan Himalayas. We present results for fall migrations for 2009, 2010, 2011 and return migrations for 2011 and 2012. We documented the existence of a complex partial altitudinal migration system. Our results offer partial support for the arrival time hypothesis. Within males, we refute the body size and the thermal tolerance hypotheses. We discuss implications of our findings within the broader context of helping understand altitudinal and partial migration.

## Results

We tagged 38 birds over three years (2009, 2010, 2011). We obtained complete downward migration data for 14 birds, return migration data for 5 birds, and data for 10 birds that did not migrate. We lost 14 birds. See Table 1 and Table S1. All data have been archived at Movebank.org.

**Table 1:** Number of individuals by year and sex classified as migrants and residents and birds for which data could not be obtained.

	No of Birds				
Year Trapped	Total Birds Tagged	Data for Fall Migration	Data for Spring/Return Migration	Data for Sedentary Birds	Not Determined/Birds Lost/
2009	10 (4F, 6M)	2 (1F, 1M)	Na	2 (1F, 1M)	6 (2F, 4M)
2010	14 (7F, 7M)	7 (5F, 2M)	0	1 (0F, 1M)	6 (2F, 4M)
2011	14 (4F, 10M)	5 (3F, 2M)	3 (2F, 1M)	7 (1F, 6M)	2 (0F, 2M)
2012	Na	Na	2 (2F,0M)	Na	Na
Total	38 (15F, 23M)	14 (9F, 5M)	5 (4F, 1M)	10 (2F, 8M)	14 (4F, 10M)

### Patterns of Migration

**Figure 1:**
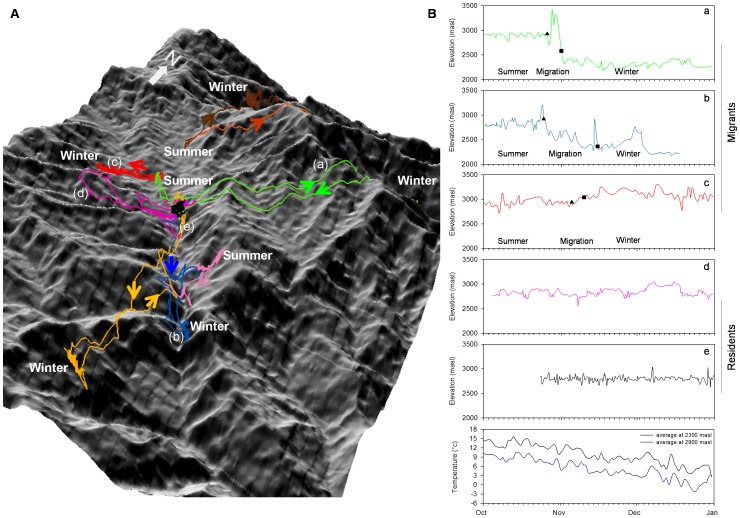
Migration patterns for 8 individual (different colours) tragopans (A). Arrows show direction of movement, and ‘Summer’ and ‘Winter’ denote summer breeding and wintering grounds. Elevation profiles (B) for 5 tragopans showing the initiation of migration (closed triangle) and end of migration (closed square). Individuals are identified by small letters ‘a’, ‘b’, ‘c’, ‘d’, ‘e’ on both (A) and (B). Temperature profiles are for October to December 2009 and January 2010 at 2300 (black line) and 2900 (blue line) masl.

**Figure 2:**
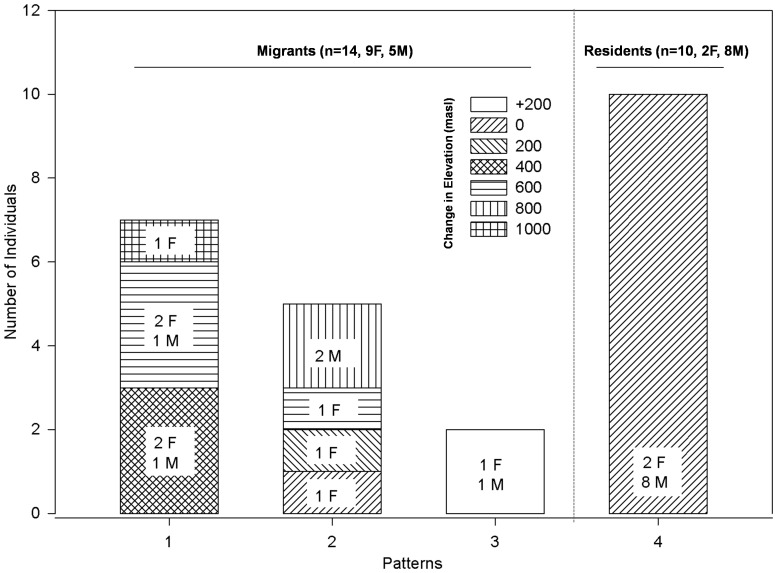
No of individuals classified as migrants or residents (Pattern 4). Migrants have been further classified into those crossing multiple mountains (Pattern 1), descending longitudinally (Pattern 2) [i.e. travelling parallel to mountain ridges], and those climbing to higher elevations in winter (Pattern 3). Hatches indicate elevation change by individuals during fall migration. ‘+200’ denotes individuals who climbed higher in winter.

We determined 3 main patterns of migration (Figure 1 and 2). Of the 14 migrants, five (3 females and 2 males) descended and returned longitudinally along mountain slopes (i.e., traveled parallel to the mountain ridges). Seven birds (5 females and 2 males) crossed over multiple mountain passes to and from their wintering grounds. Surprisingly, two birds (1 female and 1 male) actually migrated to higher elevations during winter and later returned to their breeding grounds at lower elevations.

### Who Migrates?

**Figure 3:**
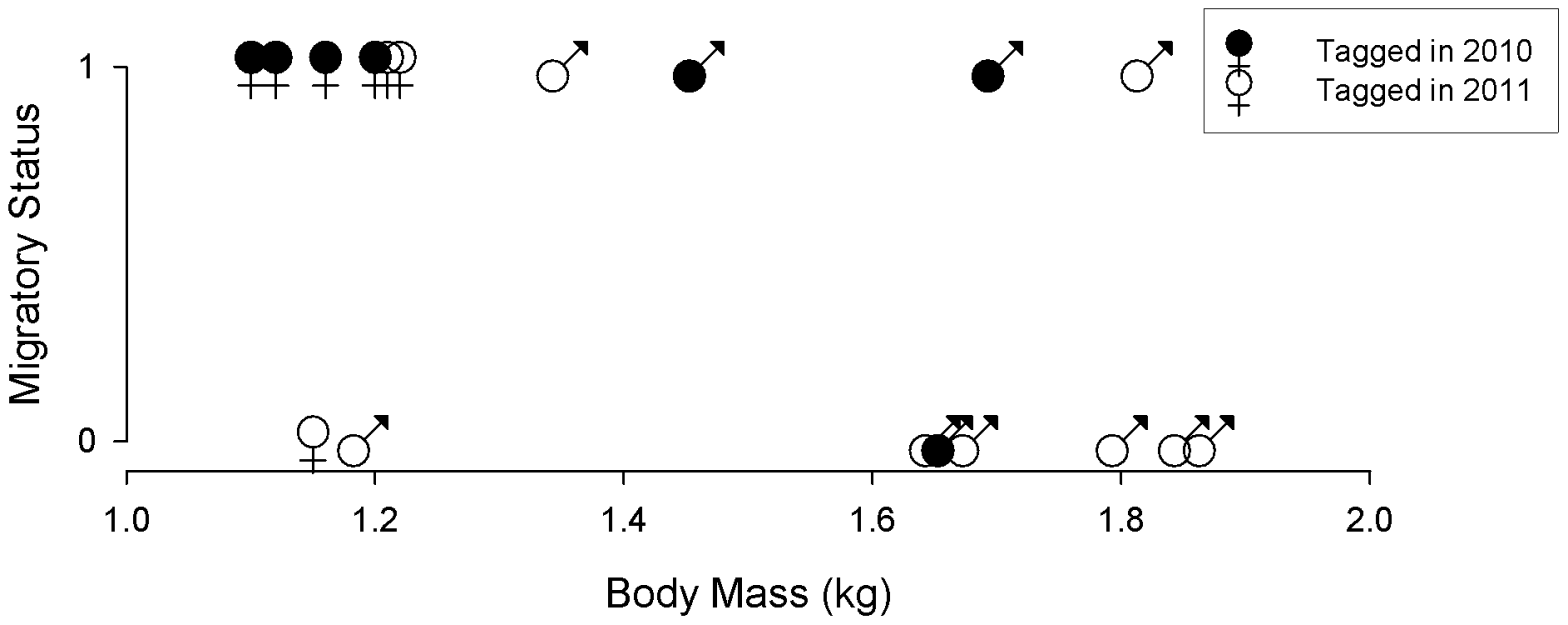
Relationship of body mass (kg) by sex to migratory status, where ‘0’ is sedentary and ‘1’ is migratory. Filled symbols represent birds tagged in 2010 and open symbols show birds tagged in 2011.

Females were significantly more likely to migrate than males (n = 24, Fisher's exact test, p = 0.047) and males were significantly heavier than females (n = 20, t = –9.8707, p = 0.000). Within males (n = 11, 4 migrants and 7 non-migrants), neither body mass nor an index of body size [body mass (kg)/tarsus length (mm)] were significant predictors for male migratory status (Table 2 and Figure 3).We did not carry out a similar analysis for females (n = 9) as we had body mass data for only one sedentary female (Figure 3).

**Table 2:** Model estimates for effect of body mass and body size index (mass/tarsus length) on migratory status of males.

Model	Std. Error	z value	Pr(>|z|)
Migratory Status ∼ Body Mass (kg)	3.004	−0.874	0.382
Migratory Status ∼ Body Mass (kg)/Tarsus Length (mm)	230.307	−0.737	0.461

### Distance and Duration of Migration

**Figure 4:**
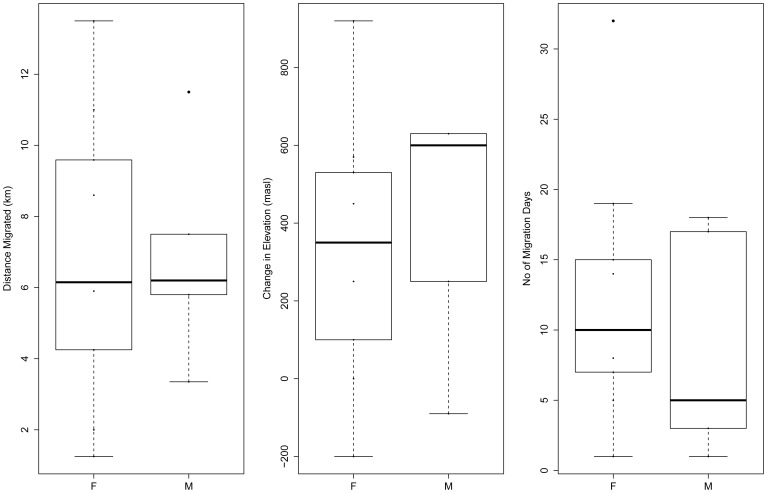
Boxplots for distance migrated (A), change in elevation (B) and duration of migration (C) by sex (n = 24, females  = 11 and males  = 13).

Duration of fall migration did not differ between sexes (n = 14, t = −0.7461, p = 0.4739) and ranged from 1 day (for an individual descending down a mountain) to 32 days (for an individual who crossed multiple mountains) with a mean of 12 (± 7.07) days for females and 8.8 (± 10.02) days for males. Distance migrated ranged from 1.25 km to more than 13.5 km and did not differ between sexes (n = 14, t = −0.0238, p = 0.9815) with a mean distance of 6.91 (±3.16) km for females and 6.87 (±3.71) km for males. Elevation differences between summer and winter grounds ranged from a gain of 920 masl (individuals descending to lower altitudes) to a loss of 190 masl (individuals ascending to higher elevation sites). There was no elevation change for one female migrant. We found no difference between males and females in terms of elevation change (n = 14, t = −0.4071, p = 0.6937). See Figure 4.

Out of the 5 birds (1 female and 4 males) for which we obtained data for return migration, 4 individuals (3 females and 1 male) returned within 4 days with one female taking upto 10 days. All returning birds displayed fidelity to their breeding sites.

### Timing of Migration

**Figure 5:**
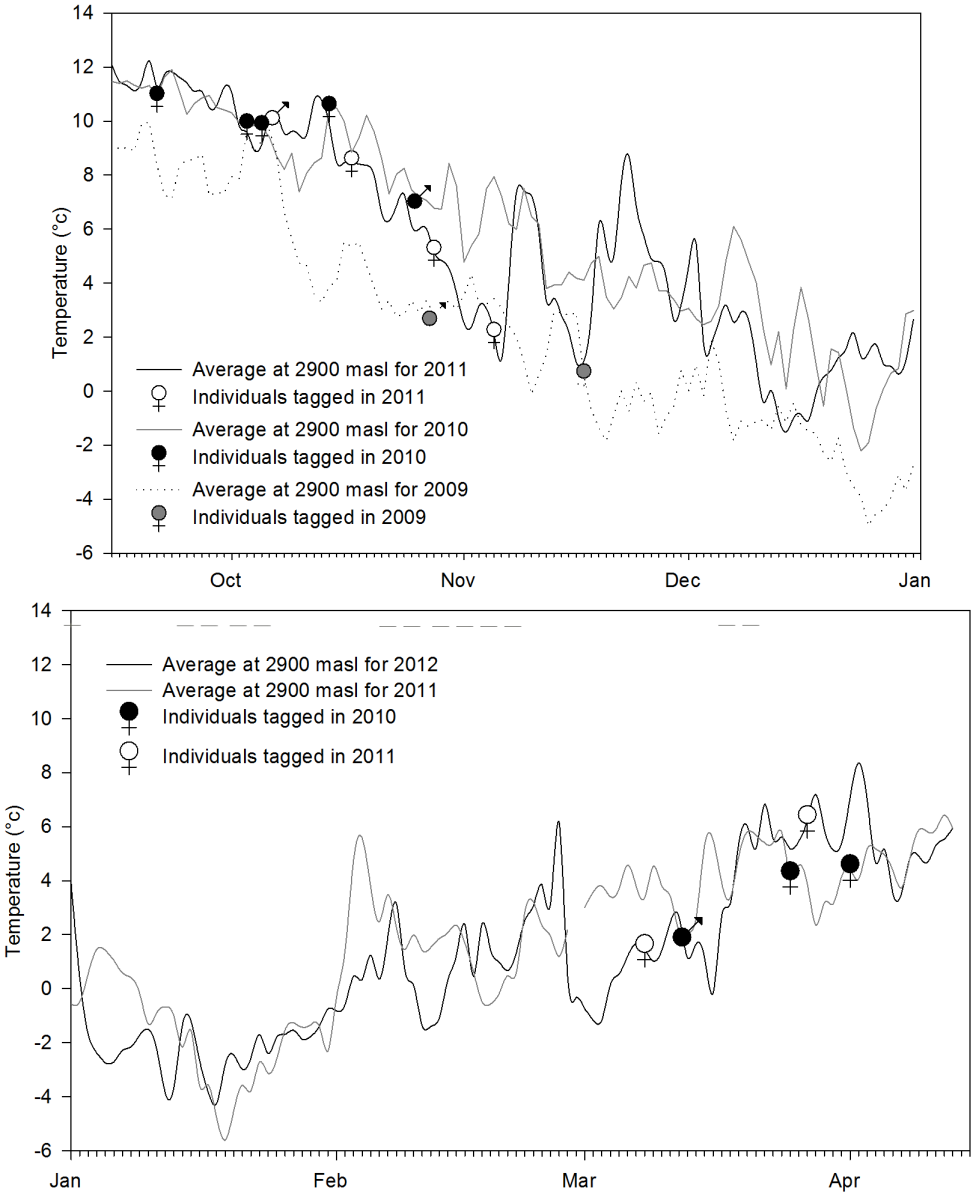
Departure dates for fall migrations for 2009, 2010 and 2011 (A) and return migrations for 2011 and 2012 (B) against temperature profiles. Dashed horizontal grey lines (B) show snowfall days in the study area.

Birds began their fall migration as early as the 3^rd^ week of September with some leaving as late as the 3^rd^ week of November (Figure 5a) with a median departure date of 26^th^ October (n = 14) for all years combined. While average daily temperatures at our study site decline gradually after August, departure dates were much ahead of snowfall events in the area (Figure 5b) which occur starting from late December and lasts till mid-March. Birds started to return between the first week of March and the first week of April following increasing temperatures beginning in mid-February (Figure 5b).

### Other Observations: Do Migrants Remain Migrants?

In 2011, we recaptured one male bird which had been tagged in 2010. While it migrated in 2010, it did not do so in 2011. We found that the bird had gained weight (2010 = 1.46 kg; 2011 = 1.68 kg), increased its beak size (2010 = 15 mm; 2011 = 16 mm) and also increased the length of its tarsus (2010 = 63 mm; 2011 = 74 mm).

## Discussion

To our knowledge, this is the first time an altitudinal migration system has been documented with high resolution GPS telemetry in the Himalayas. By tracking individuals throughout an annual migratory cycle, we confirmed that tragopans are partial altitudinal migrants. We show that migrants moving to lower/higher elevations do so by either traversing parallel to mountain ridges or by crossing multiple mountain passes. Unexpectedly, we also found that migrants move up to higher elevations in winter. Blue grouse [15] have also been found to move higher up during winters. Our results challenge the conventional notion that altitudinal migrants move to lower elevations during winter and vice-versa. Also, the complex movement patterns demonstrate that altitudinal migration is not a simple up and down-slope movement.

Our clear female biased migration system lends support to the arrival time hypothesis [34], [44] which predicts that territorial males are less likely to migrate so as to maintain their territories for the coming breeding season. However, while the hypothesis further predicts that the sex which maintains territories (i.e. males in our case) should travel shorter distances than the one that do not maintain territories (in our case, females); we found no difference between migratory males and females in terms of distance traveled or elevation changes between summer and winter. Other studies investigating the arrival time hypothesis have produced inconsistent patterns. For example, male Blue Grouse (*Dendragapus obscurus*) [15] moved farther than females, whereas female Spruce Grouse (*Canachites canadensis*) [45], [46] traveled farther than males. One way of testing the arrival time hypothesis has been the measurement of return dates to breeding sites where it is predicted that the sex which maintains territories will arrive earlier. While we found no return migration date differences between males and females, we refrain from making interpretations given our small sample sizes (4 females, 1 male). Others have reported that male Spruce Grouse [45] and Blue Grouse (as cited in [45]) return earlier to their breeding grounds. Additional data on spring arrival dates would enable us to better test this hypothesis.

Given that females are significantly smaller than males, our finding that more females migrated than males also provides support for the body-size hypothesis, which predicts that larger individuals are less likely to migrate. However, a within male investigation finds no support for this hypothesis with both smaller and lager males being equally likely to remain sedentary or to migrate. Also, in addition to smaller individuals remaining resident year round; a migrant male (1.35 kg) climbed to higher elevations (with presumably colder temperatures) in winter. Also, a female migrant (1.12 kg) climbed to higher elevations in winter. As such, we believe that individuals do not migrate as a consequence of dropping temperature levels and that body size does not determine migrants at the intra-sexual level. Our findings reinforces the need for greater consideration of inter- and intra-sexual differences while testing current hypotheses and we support the need to assess these hypotheses in cases where sexes are of the same size or where females are bigger than males [26].

We did not investigate food availability in our study system. However food availability has been important in determining upward return migrations (but not the downward fall migrations) for the White-ruffed Manikins (*Corapipo altera*) [19]. In other pheasant species, it has been noted that food may not be an important factor in driving migrations [15], [46]. A closer examination of diet preferences, individual foraging strategies, and fluctuations in food availability at both breeding and wintering sites will help clarify the role of food further.

Given that weather determines both food availability and individual thermoregulation, it has been suggested that extreme weather related events [22] drive altitudinal migrations. Our tragopan migrants departed consistently across years after rainfall peaks (July to August) and much ahead of the onset of snow (Figure 5b). As such, we believe that altitudinal migration in our case is not driven by extreme weather events.

All birds for which we have return data displayed site fidelity. These return sites were also in close proximity to sites that were occupied year-round by resident birds. Our field observations suggest that these are preferred breeding areas. As such, we speculate that density of individuals at summer breeding grounds [47]–[49] may be influencing migration in relation to the carrying capacity of breeding grounds. Further analysis on movement and activity patterns between migrants and non-migrants may help explain trade-offs in a partial migration system.

Interestingly, one male switched from being a migrant in 2010 to a non-migrant the following year. This provides anecdotal support that migration may be a plastic phenotypic response, where environmental variation can maintain differences in individual strategies [50]. This is contrary to Spruce Grouse where migratory strategies do not change [45]. Assessing the repeatability of migratory strategy over the lifetime of an individual may help further clarify this question and provide much needed answers to help address gaps in our current understanding on the role of, and balance between, genetic and environmental influences [51] in partial migration systems.

The ultimate reasons for why some individuals migrate while others remain sedentary are unclear [26]. However, we provide the first tests for a few of the existing hypotheses in a previously unstudied altitudinal migrant from the Himalayas, an important yet relatively understudied part of the world. Our observations of migrants traversing over multiple mountain ridges and even of others climbing to higher elevations is incredibly interesting, but further complicates an already puzzling phenomenon. We highlight that existing hypotheses will benefit from considering how best to explain inter- as well as intra-sexual differences. Most importantly, having shown that the patterns of an altitudinal migration system are complex and not a simple up and down slope movement, we hope our findings will influence the way altitudinal migrations are perceived and thereby contribute to a better understanding of how species may respond to climate change.

## Materials and Methods

### Study Area

**Figure 6:**
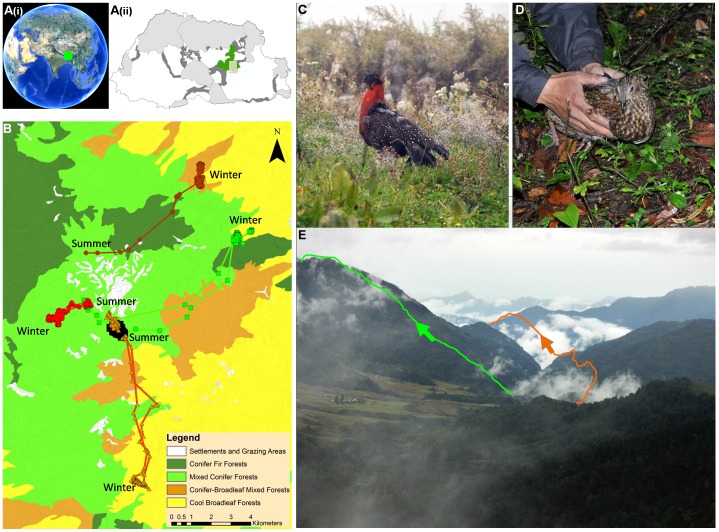
Location of Bhutan (A[i]) and Thrumshingla National Park in Bhutan shown in green (A[ii]). Location of study area bounded by rectangular box (A[ii]). Light grey areas show protected areas in Bhutan and dark grey areas show biological corridors. Land cover, locations for 1 resident (black circles) and migratory routes for 4 migratory individuals (3 females and 1 male [green squares and line] within the study area (B)). A tagged male (C) and a tagged female (D) being released. Travel route of a male migrant shown in green and female migrant shown in yellow (B) overlaid onto a photograph of the actual mountain location (E).

Tragopans were studied in Thrumshingla National Park (Figure 6) of Bhutan (27° 22′46′′ N, 91°01′46′′E). Elevation in the study area ranged from 1500 masl to 4500 masl and temperatures ranged from a maximum of 25°C to a minimum of −8°C. The area has four distinct seasons with most rainfall occurring between the months of May to August as part of the Asian monsoons. The study area is mostly conifer forests dominated by fir (*Abies densa*) with rhododendron understory at higher elevations (>3000 masl) transiting to mixed conifer forests (2400 – 3000 masl) comprising of spruce (*Picea spinulosa*), hemlock (*Tsuga dumosa*) and larch (*Larix griffithii*). Below 2400 masl, conifer forests give way to conifer-broadleaf mixed forests, and to cool broadleaved forests comprising mostly of oak (*Quercus glauca* and *Q. lamellosa*). There are also a few patches of open grazing areas used by nomadic cattle herders in the region.

### Study Species

The Satyr tragopan (*Tragopan satyra*) is a pheasant species endemic to the central and eastern Himalayas covering the countries of Nepal and Bhutan. They are also found in the state of Arunachal Pradesh in India, and some lower valleys of Xizang in China [52]. Only an estimated 20,000 individuals (about 6000 – 15000 adults) are extant in the wild [53]. The tragopans are classified as Near Threatened by the IUCN [54] and listed on Appendix III of CITES (www.cites.org). Such listings while important may not adequately reflect the actual threat to a species. In many parts of its range, it has been suggested that the tragopans face increasing threats from habitat loss, forest fires and poaching [53].

Adult male tragopans weigh from 1.3 to 2.1 kgs, while females weigh from 1 to 1.3 kgs. They are omnivorous and feed on seeds, fresh leaves, moss, bamboo shoots, berries and insects [53]. Adults perform elaborate courtship displays and breeding starts from April and lasts till June. About 3–5 eggs are laid per clutch which are then incubated for about 28 days [53]. Little is known of the biology of tragopans in the wild, and although it has been noted that tragopans are altitudinal migrants [43], no studies so far have investigated this phenomenon.

We trapped tragopans in 2009, 2010 and 2011 using neck noose traps laid along known haunts following ridges which we barricaded with bamboo and other shrub species. We flushed tragopans towards traps during early mornings and evenings. All animal trapping were approved by the Ministry of Agriculture and Forests in Bhutan. In 2009, in order to reduce any handling related fatality given that these pheasants were trapped for the first time, all captured pheasants were released immediately after attaching GPS tags. Pheasants captured in 2010 and 2011 were weighed (to the nearest gm) and measurements were also taken of tarsus length (mm) and beak size (mm).

### GPS Tags and Data Acquisition

We used GPS/accelerometer tags (www.e-obs.de, Munich, Germany) to record the location (GPS) and activity (accelerometer) of our tragopans. Tags with harnesses weighed 45 gms. These tags save the recorded data (i.e., location, elevation, date, time and acceleration) onboard to be remotely downloaded via a handheld base station after the tragopan is relocated via the tag VHF radio pulse (ping). To help locate tagged birds, tags were programmed to ping once every 2 seconds for 2 hours every day.

Tags deployed in 2009 were programmed to take a GPS reading every 2 hours from 0400 hrs to 2200 hrs. Given battery power constraints; tags in 2010 were programmed to take only 2 GPS readings everyday at 0600 hrs and 1400 hrs; while in 2011 tags were programmed to take 3 GPS readings everyday at 0800 hrs, 1400 hrs and 2000 hrs. In order to optimize battery performance, tags were further programmed with GPS ‘give up times’ of 2 minutes, after which the tag does not try to obtain a GPS fix for that particular location.

### Distance and Duration of Migration

We classified all birds which showed distinct summer and winter ranges (birds staying more than a month at a given location) as migrants and the rest as residents. We measured migration distance using the ‘show elevation profile’ tool in Google Earth 5.2 as the distance between the location on the day when migration was initiated to the first location of the day when migration was terminated. Total number of migration days was calculated as difference between the date of initiation of migration and the date of cessation of migration.

### Temperature Profile

Temperature loggers (HOBO ©) were placed at 2900 masl (summer range) and 1700 masl (assumed winter range) during 2009. In 2010, after ascertaining winter ranges for 2 migrating birds, additional loggers were deployed at 2700 masl and 2300 masl. Loggers were programmed to record temperatures averaged across every 20 minutes at a sampling interval of 10 seconds. Gain in temperatures were calculated as the difference between the average temperature at 2900 masl on the day when migration was initiated and the temperature at 2300 masl on the day when migration was terminated.

### Statistical Analyses

We used body mass (kg) and a body size index (mass [kg]/tarsus length [mm]) and developed a logistic regression model with a logit link function in R (http://www.r-project.org; version 2.15.1) to test whether an individual's migratory status was related to its body mass and size index. All other statistical tests were also performed in R.

## Supporting Information

Table S1 **Details of tagged individuals showing date of deployment, last day of GPS fix and fix rates per day.**
(DOCX)

## References

[1] Dingle H, Drake VA (2007) What is migration? BioScience 57: 113–122.

[2] Wilcove DS, Wikelski M (2008) Going, going, gone: is animal migration disappearing. PLoS Biol 6: e188. 18666834 10.1371/journal.pbio.0060188 PMC2486312

[3] Dingle H (1996) Migration: The Biology of Life on the Move. New York:Oxford University Press.

[4] Bowlin MS, Bisson I, Shamoun-baranes J, Reichard JD, Sapir N, et al (2010) Grand challenges in migration biology. Integrative and Comparative Biology 50(3): 261–279 doi:10.1093/icb/icq013. 10.1093/icb/icq013 PMC7108598 21558203

[5] Schwenk K, Padilla DK, Bakken GS, Full RJ (2009) Grand challenges in organismal biology. Comparative and General Pharmacology 49: 7–14 doi:10.1093/icb/icp034. 10.1093/icb/icp034 21669841

[6] Wilcove DS (2008) Animal migration: an endangered phenomenon? Issues in Science and Technology 24(3): 71–79.

[7] Wilcove D (2008) No Way Home: The Decline of the World's Great Animal Migrations. Washington,D.C.:Island Press.

[8] Newton I (2008) The Migration Ecology of Birds. London:Academic Press.

[9] Berthold P (2001) Bird Migration: A General Survey. New York: Oxford University Press.

[10] Stiles FG (1988) Altitudinal movements of birds on the Caribbean slope of Costa Rica: Implications for conservation. In: Alameda F, Pringle CM, editors.Tropical Rainforests: Diversity and Conservation.San Francisco:California Academy of Sciences. pp. 243–258.

[11] Hahn TP, Sockman KW, Breuner CW, Morton ML (2004) Facultative altitudinal movements by mountain white-crowned sparrows (*Zonotrichia leucophrys oriantha*) in the Sierra Nevada. The Auk 121: 1269–1281.

[12] Laymon SA (2010) Altitudinal migration movements of Spotted Owls in the Sierra Nevada, California. Sierra 91: 837–841.

[13] Powell GVN, Bjork R (1995) Implications of intratropical migration on reserve design: Case study using Pharomachrus mocinno. . Conservation Biology 9: 354–362.

[14] Chaves-Champos J, Arevalo JE, Araya M (2003) Altitudinal movements and conservation of Bare-necked Umbrellabird (*Cephalopterus glabricollis*) of the Tilaran Mountains, Costa Rica. Bird Conservation International 13: 45–58 doi:10.1017/S0959270903003046.

[15] Cade BS, Hoffman RW (1993) Differential migration of Blue Grouse in Colorado. The Auk 110: 70–77.

[16] Mackas RH, Green DJ, Whitehorne IBJ, Fairhurst EN, Middleton HA, et al (2010) Altitudinal migration in American Dippers (*Cinclus mexicanus*): Do migrants produce higher quality offspring? Canadian Journal of Zoology 88: 369–377.

[17] Dixon KL, Gilbert JD (1964) Altitudinal migration in the Mountain Chickadee. The Condor 66: 61–64.

[18] Blake JG, Stiles FG, Loiselle BA (1993) Birds of La Selva Biological Station: habitat use, trophic composition, and migrants. In:, A Gentry, editor. Four Neo-tropical Rain Forests.New Haven :Yale University Press. pp. 161–182.

[19] Boyle WA (2010) Does food abundance explain altitudinal migration in a tropical frugivorous bird? Canadian Journal of Zoology 88: 204–213.

[20] Loiselle BA, Blake JG (1991) Temporal variation in birds and fruits along an elevational gradient in Costa Rica. Ecology 72: 180–193.

[21] Boyle WA, Conway CJ, Bronstein JL (2011) Why do some, but not all, tropical birds migrate? A comparative study of diet breadth and fruit preference. Evolutionary Ecology 25(11): 219–236 doi:10.1007/s10682-010-9403-4.

[22] Boyle WA, Norris DR, Guglielmo CG (2010) Storms drive altitudinal migration in a tropical bird. Proceedings of the Royal Society 277: 2511–2519 doi:10.1098/rspb.2010.0344. 10.1098/rspb.2010.0344 PMC2894928 20375047

[23] Boyle WA (2008) Can variation in risk of nest predation explain altitudinal migration in tropical birds? Oecologia 155(2): 397–403 doi:10.1007/s00442-007-0897-6. 18188606 10.1007/s00442-007-0897-6

[24] Hess SC, Leopold CR, Misajon K, Hu D, Jeffrey JJ (2012) Restoration of movement patterns of the Hawaiian Goose. The Wilson Journal of Ornithology 124: 478–486.

[25] Powell GVN, Bjork RD (2010) Implications of altitudinal migration for conservation strategies to protect tropical biodiversity: a case study of the Resplendent Quetzal (*Pharomacrus mocinno*) at Monteverde, Costa Rica. Bird Conservation International 4: 161–174.

[26] Chapman B, Brönmark C, Nilsson J, Hansson L (2011) The ecology and evolution of partial migration. Oikos 120: 1764–1775.

[27] Chapman BB, Brönmark C, Nilsson J-Å, Hansson L-A (2011) Partial migration: an introduction. Oikos 120: 1761–1763 doi:10.1111/j.1600-0706.2011.20070.x.

[28] Shaw AK, Levin SA (2011) To breed or not to breed: a model of partial migration. Oikos 120: 1871–1879 doi:10.1111/j.1600-0706.2011.19443.x.

[29] Boyle W (2008) Partial migration in birds: tests of three hypotheses in a tropical lekking frugivore. Journal of Animal Ecology 77: 1122–1128. 18657208 10.1111/j.1365-2656.2008.01451.x

[30] Boyle WA, Guglielmo CG, Hobson KA, Norris DR (2011) Lekking birds in a tropical forest forego sex for migration. Biology Letters 7: 661–663. 21471048 10.1098/rsbl.2011.0115 PMC3169044

[31] Cristol DA, Baker MB, Carbone C (1999) Differential migration revisited: latitudinal segregation by age and sex class. In: Nolan VJ, Ketterson ED, Thompson CF, editors. Current Ornithology.Kluwer Academic. pp. 33–88.

[32] Bell CP (2005) Inter- and intrapopulation migration patterns: ideas, evidence and research priorities. In: Greenberg R, Marra PP, editors. Birds of Two worlds: The Ecology and Evolution of Migration.The John Hopkins University Press. pp. 41–52.

[33] Gauthreaux SA (1978) The ecological significance of behavioural dominance. Perspectives in Ethology 71: 17–54.

[34] Ketterson ED, Nolan Jr V (1976) Geographic variation and its climatic correlates in the sex ratio of eastern-wintering Dark-Eyed Juncos (*Junco Hyemalis Hyemalis*). Ecology 57: 679–693.

[35] Ketterson E, Nolan Jr V (1983) The evolution of differential bird migration. Current Ornithology I: 357–402.

[36] Alonso J, Palacín C, Alonso J, Martín C (2009) Post-breeding migration in male great bustards: low tolerance of the heaviest Palaearctic bird to summer heat. Behavioral Ecology and Sociobiology 63: 1705–1715.

[37] Calder WA (1974) Consequences of body size for avian energetics. Avian Energetics 15: 86–151.

[38] Lundberg P (1985) Dominance behaviour, body weight and fat variations, and partial migration in European blackbirds (*Turdus merula*). Behavioral Ecology and Sociobiology 17: 185–189.

[39] Jahn AE, Levey DJ, Hostetler JA, Mamani AM (2010) Determinants of partial bird migration in the Amazon Basin. The Journal of Animal Ecology 79: 983–992 doi:10.1111/j.1365-2656.2010.01713.x. 20546065 10.1111/j.1365-2656.2010.01713.x

[40] Nogués-Bravo D, Araújo MB, Errea MP, Martínez-Rica JP (2007) Exposure of global mountain systems to climate warming during the 21st Century. Global Environmental Change 17: 420–428.

[41] Faaborg J, Holmes RT, Anders AD, Bildstein KL, Dugger KM, et al (2010) Recent advances in understanding migration systems of New World land birds. Ecological Monographs 80: 3–48.

[42] Inouye DW, Barr B, Armitage KB, Inouye BD (2000) Climate change is affecting altitudinal migrants and hibernating species. Proc Natl Acad Sci USA 97: 1630–1633. 10677510 10.1073/pnas.97.4.1630 PMC26486

[43] Grimmett R, Inskipp C, Inskipp T (1999) Birds of India, Pakistan, Nepal, Bangladesh, Bhutan, Sri Lanka, and the Maldives. Princeton, New Jersey:Princeton University Press.

[44] Morbey Y, Coppack T, Pulido F (2012) Adaptive hypotheses for protandry in arrival to breeding areas: a review of models and empirical tests. Journal of Ornithology 153: 207–215.

[45] Herzog PW, Keppie DM (1980) Migration in a local population of Spruce Grouse. The Condor 82: 366–372.

[46] Schroeder MA, Braun CE (1993) Partial migration in a population of Greater Prairie-Chickens in Northeastern Colorado. The Auk 110: 21–28.

[47] Lundberg P (1987) Partial bird migration and evolutionarily stable strategies. Journal of Theoretical Biology 125: 351–360.

[48] Lundberg P (1988) The evolution of partial migration in birds. TREE 3: 172–175. 21227194 10.1016/0169-5347(88)90035-3

[49] Taylor CM, Norris DR (2007) Predicting conditions for migration: effects of density dependence and habitat. Biology Letters 3: 280–284 doi:10.1098/rsbl.2007.0053. 17374588 10.1098/rsbl.2007.0053 PMC2464694

[50] Brodersen J, Nilsson PA, Chapman BB, Skov C, Hansson L-A, et al (2011) Variable individual consistency in timing and destination of winter migrating fish. Biology Letters 8: 21–23. 21813551 10.1098/rsbl.2011.0634 PMC3259970

[51] Pulido F (2011) Evolutionary genetics of partial migration – the threshold model of migration revisited. Oikos 120: 1776–1783 doi:10.1111/j.1600-0706.2011.19844.x.

[52] Sibley CG, Monroe BL (1990) Distribution and Taxonomy of Birds of the World. New Haven,USA:Yale University Press.

[53] BirdLife International (2012) Species factsheet: Tragopan satyra. Available:http://www.birdlife.org.Accessed 2 November 2012.

[54] IUCN (2008) IUCN Red List. Available: http://www.iucnredlist.org.

