# Supplementary material for: Partial Altitudinal Migration of a Himalayan Forest Pheasant
Source: PLoS One. 2013 Apr 26;8(4):e60979. doi: 10.1371/journal.pone.0060979 (PMC3637220; doi:10.1371/journal.pone.0060979)
Supplement: Table S1 — Details of tagged individuals showing date of deployment, last day of GPS fix and fix rates per day. (DOCX) [file pone.0060979.s001.docx]

| Tag ID | Year Tagged | Sex | Weight (kg) | Migratory Status | Tag Deployed On | Date of Last GPS Fix | No of Days with Data | Total GPS Fixes | GPS Fix/Day | Comments |
| --- | --- | --- | --- | --- | --- | --- | --- | --- | --- | --- |
| 736 | 2009 | M | -- | U^1^ | 7/3/2005 |  |  |  |  | Lost |
| 737 | 2009 | M | -- | U | 6/30/2005 | 10/23/2005 | 113 | 319 | 2.8 | Dead |
| 743 | 2009 | F | -- | U | 7/25/2005 | 9/7/2005 | 42 | 122 | 2.9 | Dead |
| 744 | 2009 | F | -- | U | 8/22/2005 | 8/23/2005 | 1 | 2 | 2.0 | Lost |
| 748 | 2009 | M | -- | S^2^ | 6/3/2005 | 12/12/2005 | 189 | 1135 | 6.0 |  |
| 749 | 2009 | M | -- | U | 6/30/2005 | 10/21/2005 | 111 | 222 | 2.0 | Dead |
| 750 | 2009 | F | -- | M^3^ | 8/22/2005 | 11/30/2005 | 98 | 518 | 5.3 |  |
| 753 | 2009 | M | -- | M | 8/20/2005 | 12/21/2005 | 121 | 566 | 4.7 |  |
| 758 | 2009 | M | -- | U | 7/4/2005 | 10/22/2005 | 108 | 619 | 5.7 | Dead |
| 759 | 2009 | F | -- | S | 6/4/2005 | 11/21/2005 | 167 | 479 | 2.9 |  |
| 1276 | 2010 | M | 1.66 | S | 9/9/2006 | 4/22/2007 | 223 | 205 | 0.9 |  |
| 1277 | 2010 | M | 1.82 | U | 9/21/2006 | 9/23/2006 | 2 | 2 | 1.0 | Lost |
| 1278 | 2010 | M | 1.7 | U | 9/21/2006 | 9/21/2006 | 1 | 1 | 1.0 | Lost |
| 1279 | 2010 | F | 1.1 | M | 9/16/2006 | 3/15/2007 | 179 | 204 | 1.1 |  |
| 1281 | 2010 | M | 1.64 | U | 9/19/2006 | 9/24/2006 | 5 | 5 | 1.0 | Lost |
| 1282 | 2010 | M | 1.46 | M | 9/6/2006 | 3/3/2007 | 177 | 171 | 1.0 |  |
| 1283 | 2010 | F | 1.2 | U | 9/8/2006 | 9/15/2006 | 7 | 11 | 1.6 | Lost |
| 1284 | 2010 | F | 1.16 | M | 9/19/2006 | 3/7/2007 | 168 | 180 | 1.1 | Dead |
| 1415 | 2010 | F | 1.12 | M | 9/23/2006 | 4/3/2007 | 190 | 189 | 1.0 |  |
| 1416 | 2010 | F | 1.2 | M | 9/24/2006 | 3/18/2007 | 174 | 170 | 1.0 |  |
| 1417 | 2010 | F | 1.16 | M | 9/21/2006 | 4/20/2007 | 209 | 277 | 1.3 |  |
| 1418 | 2010 | F | 1.29 | U | 9/22/2006 | 10/28/2006 | 36 | 45 | 1.3 | Dead |
| 1419 | 2010 | M | 1.7 | M | 9/22/2006 | 3/16/2007 | 174 | 141 | 0.8 |  |
| 1421 | 2010 | M | 1.77 | U | 9/22/2006 | 10/28/2006 | 36 | 48 | 1.3 | Dead |
| 1410 | 2011 | M | 1.35 | M | 10/14/2007 | 3/11/2008 | 147 | 317 | 2.2 |  |
| 1411 | 2011 | M | 1.68 | S | 10/11/2007 | 9/4/2008 | 323 | 504 | 1.6 | Same as 1282 |
| 1413 | 2011 | M | 1.8 | U | 10/14/2007 |  |  |  |  | Lost |
| 1414 | 2011 | M | 1.82 | M | 9/28/2007 | 1/8/2008 | 100 | 171 | 1.7 |  |
| 1418 | 2011 | M | 1.6 | U | 9/24/2007 |  |  |  |  | Lost |
| 1894 | 2011 | M | 1.19 | S | 10/25/2007 | 4/14/2008 | 169 | 351 | 2.1 |  |
| 1895 | 2011 | F | 1.22 | M | 10/22/2007 | 9/4/2008 | 312 | 350 | 1.1 |  |
| 1896 | 2011 | F | 1.21 | M | 10/22/2007 | 7/11/2008 | 259 | 437 | 1.7 |  |
| 1897 | 2011 | F | 1.2 | M | 9/21/2007 | 4/4/2008 | 193 | 437 | 2.3 | Dead |
| 1898 | 2011 | M | 1.87 | S | 10/22/2007 | 9/4/2008 | 312 | 521 | 1.7 |  |
| 1899 | 2011 | M | 1.85 | S | 10/18/2007 | 7/17/2008 | 269 | 515 | 1.9 |  |
| 1901 | 2011 | M | 1.65 | S | 9/28/2007 | 9/4/2008 | 336 | 422 | 1.3 |  |
| 1902 | 2011 | F | 0.94 | S | 10/22/2007 | 12/11/2007 | 49 | 97 | 2.0 |  |
| 1903 | 2011 | M | 1.8 | S | 10/16/2007 | 9/4/2008 | 318 | 572 | 1.8 |  |
| 1905 | 2011 | F | 1.15 | S | 10/16/2007 | 8/8/2008 | 292 | 548 | 1.9 |  |

Supplementary Table: Details of tagged individuals showing date of deployment, last day of GPS fix and fix rates per day

1: Undetermined

2: Sedentary

3: Migrant
